# Hierarchical nanostructured aluminum alloy with ultrahigh strength and large plasticity

**DOI:** 10.1038/s41467-019-13087-4

**Published:** 2019-11-08

**Authors:** Ge Wu, Chang Liu, Ligang Sun, Qing Wang, Baoan Sun, Bin Han, Ji-Jung Kai, Junhua Luan, Chain Tsuan Liu, Ke Cao, Yang Lu, Lizi Cheng, Jian Lu

**Affiliations:** 1 Department of Mechanical Engineering, City University of Hong Kong, Hong Kong, China; 2 School of Science, Harbin Institute of Technology, Shenzhen, 518055 China; 3 Hong Kong Branch of National Precious Metals Material Engineering Research Centre, City University of Hong Kong, Hong Kong, China; 4 Laboratory for Microstructures, Institute of Materials Science, Shanghai University, Shanghai, 200072 China; 5 Institute of Physics, Chinese Academy of Sciences, Beijing, 100190 China; 6 Songshan Lake Materials Laboratory, Dongguan, Guangdong 523808 China; 7 Department of Materials Science and Engineering, City University of Hong Kong, Hong Kong, China; 8 Centre for Advanced Structural Materials, Shenzhen Research Institute of City University of Hong Kong, Shenzhen, 518057 China

**Keywords:** Mechanical properties, Metals and alloys, Structural properties

## Abstract

High strength and high ductility are often mutually exclusive properties for structural metallic materials. This is particularly important for aluminum (Al)-based alloys which are widely commercially employed. Here, we introduce a hierarchical nanostructured Al alloy with a structure of Al nanograins surrounded by nano-sized metallic glass (MG) shells. It achieves an ultrahigh yield strength of 1.2 GPa in tension (1.7 GPa in compression) along with 15% plasticity in tension (over 70% in compression). The nano-sized MG phase facilitates such ultrahigh strength by impeding dislocation gliding from one nanograin to another, while continuous generation-movement-annihilation of dislocations in the Al nanograins and the flow behavior of the nano-sized MG phase result in increased plasticity. This plastic deformation mechanism is also an efficient way to decrease grain size to sub-10 nm size for low melting temperature metals like Al, making this structural design one solution to the strength-plasticity trade-off.

## Abstract (web summary)

Strengthening a metallic alloy without sacrificing ductility remains challenging. Here, the authors develop a hierarchical nanostructured aluminium alloy composed of nanograins surrounding by metallic glass shells that has both ultrahigh strength and good ductility.

## Introduction

Stronger crystalline alloys are usually designed by controlling defects to hinder dislocation motion. These defects can be classified as point, line, interface, and volume defects^1^. Correspondingly, the mechanisms^1,2^ for making materials stronger are solid solution strengthening, dislocation strengthening, grain (or interphase) boundary strengthening, and precipitate (or dispersed reinforcement particle) strengthening. Strengthening approaches usually decrease ductility as a compromise^2^. In order to overcome this strength-ductility trade-off, several strategies have been developed. For example, certain twin boundaries (TBs) allow dislocations to move in neighboring domains (twin or matrix) or glide along TBs^3^ and phase transformation induced plasticity (TRIP) effect may provide strain hardening^4^, thereby enhancing ductility. In contrast, non-crystalline solids do not possess slip systems and lattice dislocations due to the lack of long-range periodicity in their atomic structures^5^. Thus, amorphous metallic glasses (MGs) have quite different deformation mechanisms when compared with their crystalline counterparts^6^. In absence of dislocation-mediated crystallographic slip, MGs manifest large elastic deformation of 2% prior to yielding and correspondingly exhibit high yield strength superior to crystalline alloys^6^. For example, the compressive strength of Co-based MGs^7^ can reach ~5 GPa and many Fe-based MGs^8^ show strength as large as ~4 GPa. However, plastic deformation of MGs at ambient temperature is highly localized in shear bands^9^, thus leading to a catastrophic failure without any significant macroscopic ductility. Through heterogeneity nanostructuring^10^ or crystalline phase addition^11^/transformation^12,13^, the ductility of MGs can be enhanced. The ductility can be increased to ~7% after introducing a B2 phase^13^ into the MG matrix. By amorphous/crystalline laminate nanostructuring, the plastic deformation can be increased to 10~30%^14–17^. In these approaches, plasticity originates from the dislocation movement inside the crystalline phase and multiple shear bandings in the MG phase. However, the strength of these MG matrix composites (MGMC) have difficulty reaching that of their MG counterpart as a result of soft crystalline phases or the shear band softening effect, unless other strengthening mechanisms^13^ balance these softenings. If the size of the MGs is smaller than 100 nm, the shear banding event can be fully suppressed^18,19^, which contributes to an ideal strength^20^ and homogeneous plastic flow behavior of the MGs. Therefore, we hypothesize that with an extremely thin MG phase surrounding the crystalline phase, strain hardening of the crystalline phase and plastic flow of the nano-sized MG phase will contribute to both high strength and large ductility.

To realize such a material, we develop a hierarchical nanostructured Al alloy composed of face-centered-cubic (*fcc*) nanograins surrounding by nano-sized MG shells. The grain boundaries are mostly replaced by the strong MG shells, which contributes to an ultrahigh strength of 1.7 GPa. During plastic deformation, the nano-sized MG phase exhibits flow behavior due to its small size. Dislocations are initiated from the nanograin/MG interfaces. Some dislocations pile up in the nanograins and provide strain hardening, while the majority of the dislocations move within the nanograins and annihilate at another nanograin/MG interface. This deformation mechanism contributes to the large plasticity.

## Results

### Structure design aided by molecular dynamic simulations

**Fig. 1:**
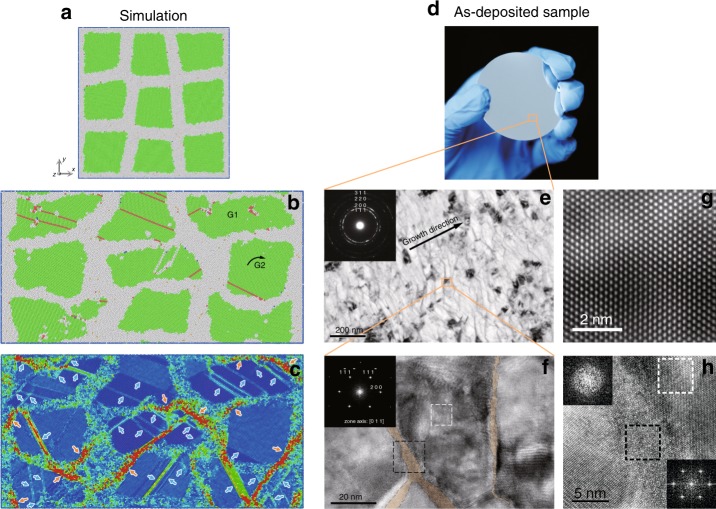
Hierarchical nanostructure guided from computer simulation. **a** The atomistic model of the glass-crystal hierarchical nanostructure composed of amorphous Al_85_Ni_15_ and Al nanograins. **b** MD simulated atomic snapshot and **c** the corresponding atomic shear strain distribution of the hierarchical nanostructured Al alloy at a strain of 30%. ‘G1’ and ‘G2’ in **b** represent a flattened ‘grain 1’ and a rotated ‘grain 2’, respectively. The typical dislocation slips in ‘G1’ contribute to plastic deformation. Large rotation with little dislocation propagation in ‘G2’ may also accommodate the plastic deformation. **d** Optical image of the hierarchical nanostructured Al alloy. **e** Cross-sectional TEM image of the hierarchical nanostructured Al alloy, showing Al nanograins surrounded with light–dark contrasted grain–grain interfaces. The selected area electron diffraction (SAED) pattern in the inset denotes the polycrystalline structure. **f** HRTEM image of the hierarchical nanostructured Al alloy, showing a crystalline Al nanograin is surrounded by amorphous phase (post-colored by light yellow). The inset is fast Fourier transformation (FFT) image of the white dashed rectangle region in the main image, showing the *fcc* structure with zone axis of [0 1 1]. **g**, **h** are the zoomed-in images of white and black dashed rectangle regions in (**f**), respectively. **g** Shows a defect-free *fcc* structure. **h** Shows a nano-sized amorphous phase forms between the two grains. The FFT image (lower right inset of **h**) of the crystalline region (white dashed rectangle) reveals pronounced spots pattern. By contrast, the FFT image (upper left inset of **h**) of the black dashed rectangle region in **h** shows a diffuse pattern, indicating the amorphous structure. The termination ripples feature may be due to the small sampling size

We conducted computer simulations to verify the hypothesis first, then a hierarchical nanostructure was built accordingly. *fcc* metals are an ideal model since the dominant mode of deformation is dislocation glide and climb. The plastic deformation of aluminum (Al) is based on this mechanism, and twinning is difficult to initiate due to its high stacking fault energy^21^. The universal *fcc* structure and the dislocation-slip-based plastic deformation mechanism led to our material design of hierarchical nanostructured Al alloy. The hierarchical nanostructure of the Al nanograins embedded in the Al_85_Ni_15_ MG was designed and then simulated by the molecular dynamic (MD) (Fig. 1a and Supplementary Fig. 1). A simulated compression test was performed on the structure. Our results show that Al nanograins become shorter and wider at a strain of 30% (Fig. 1b), without any global shear of the whole structure or cavitation generation in the local area. This uniform plastic deformation is rarely seen in nanocrystalline, amorphous^5^ or glass-crystal nanocomposite^22^ materials, which usually possess high strength yet limited plasticity. Dislocations are initiated from the glass/crystal interface, indicated by the locally largest atomic shear strain on the interface (Fig. 1c, highlighted by the orange arrow). The large atomic shear strain in the amorphous phase indicates plastic flow behavior. Meanwhile, dislocation slip dominates the plastic deformation mechanism of the Al nanograins, leaving local larger atomic shear strain trajectories (Fig. 1c, highlighted by light blue arrow). The cross slip of dislocations offset the atomic shear strain, resulting in a serious underestimation of the number of dislocations during deformation. This effect is shown in a structure evolution movie (Supplementary Movie 1) of ‘G1’ in Fig. 1b. Despite previous studies reporting that glass-crystal nano-dual-phase nanostructuring could provide ultrahigh strength yet lose plasticity^22^, that study shows the limited plasticity may be attributed to the brittle nature of intermetallic nanocrystals. By substituting the brittle intermetallic phase with a ductile *fcc* Al phase, dislocation motion in the Al phase could initiate plastic deformation in the hierarchical nanostructured Al alloy. In addition, we fabricated another hierarchical nanostructure model with much lower fraction of MG phase (~1/10 size of the crystalline phase) and which shows similar deformation behavior (Supplementary Fig. 2).

### Microstructure characterization

**Fig. 2:**
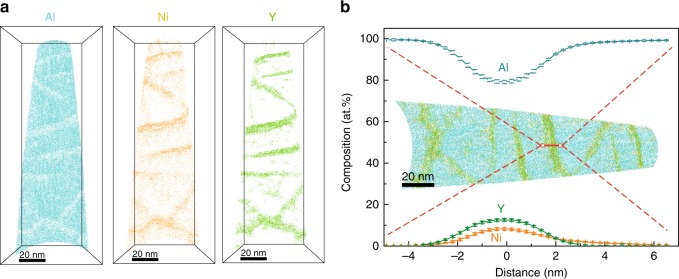
3D heterogeneity of the hierarchical nanostructured Al alloy. **a** Thin slice from three-dimensional reconstruction of an APT dataset, showing Ni and Y elements enrichment in the interface between pure Al nanograins. **b** Atom distribution showing the composition change across the selected interface. The error bars are standard deviations

The 3D hierarchical structure designed by MD simulation has shown large plasticity, and the Al alloy exhibiting such characteristic can be successfully fabricated. In former reports, the Al content is usually 80–90% (at.%) in conventional Al-based MGs^23^. If the Al content exceeds 90%, nearly pure *fcc* Al crystals could be generated in the MG matrix^24^. Therefore, we increased the Al content to 95% (at.%) in our Al-Ni-Y alloy to obtain a glass-crystal dual-phase. The average composition of the hierarchical nanostructured Al alloy is Al_95_Ni_2_Y_3_ (at.%). The *fcc* Al nanograins with a diameter of ~40 nm are dispersed uniformly across the whole specimen. The size of the nanograins was discerned from the darkest contrast regions in Fig. 1e. The structural unit of the hierarchical nanostructured Al alloy is tabular-like with ~40 nm width and ~100 nm length. Therefore, there are 2–3 nanograins in each structural unit. The interfaces between the structural units (gray lines in Fig. 1e) are not grain boundaries (GBs) as in conventional polycrystalline materials, but are a secondary amorphous phase with a thickness of ~4 nm (Fig. 1h). The maze-like pattern in the high resolution transmission electron microscope (HRTEM) image and diffuse pattern in the corresponding FFT image indicate the amorphous structure. Furthermore, to unambiguously reveal the structure difference between the amorphous nanolayer and the conventional GB, we annealed the hierarchical nanostructured Al alloy at 300 °C for 2 h to fully crystallize the amorphous phase. The HRTEM image of the grain–grain interface is shown in Supplementary Fig. 3, which demonstrates a distinct difference from the amorphous nanolayer. The amorphous phase in the as-deposited sample possesses a thickness of ~4 nm and length of tens to hundred nanometers, which is larger than the ~1 nm-sized short/medium range ordered clusters in MGs^25,26^. It is similar to an amorphous intergranular film, a type of GB complexion^27^, which is usually 1–5 nm thick^28,29^. Because the composition of this amorphous phase (Fig. 2) is a good glass former, it manifests as an MG phase.

### Element distributions in the MG phase

The hierarchical nanostructured Al alloy exhibits chemical composition heterogeneity (Fig. 2). The composition of the amorphous phase is Al_80_Ni_8_Y_12_ (at.%), and the nanograins are pure Al, which was investigated by atomic probe tomography (APT). The volume fraction of the amorphous phase is calculated to be 25% based on the compositions of the whole material (Al_95_Ni_2_Y_3_), the crystalline phase (Al_100_Ni_0_Y_0_) and the MG phase (Al_80_Ni_8_Y_12_). Furthermore, it is easy to calculate the volume fraction from the reconstruction of the APT results, which is estimated to be 20%. While Al_80_Ni_8_Y_12_ is a typical glass forming composition^23^, the amorphous Al_80_Ni_8_Y_12_ phase is formed under fast cooling condition during the fabrication process, leaving Al nanocrystals with poor glass forming ability. The alloy system is not fully amorphous because the Al content is 95%, which is beyond the glass forming region for Al-based MGs (Al content of 80–90%^27^). The formation of Al nanocrystals leads to solute rejection into the MG phase, which in turn stabilizes it. Al nanocrystals do not grow into columns as other alloy films do^30^ in the sputtering process, which could be attributed to the fact that the nano-sized MG phase acts as an interruption barrier, disrupting the preferred growth pattern of Al nanocrystals. It should be noted that the *fcc* Al phase in the Al-based MGMCs can be generated by composition control^24^ or post heat treatment of MGs^31^. However, the hierarchical nanostructure with such extremely thin MG phase reported here, which is essential for good mechanical properties, has rarely been seen in previous reports^24,31^.

### Mechanical properties

**Fig. 3:**
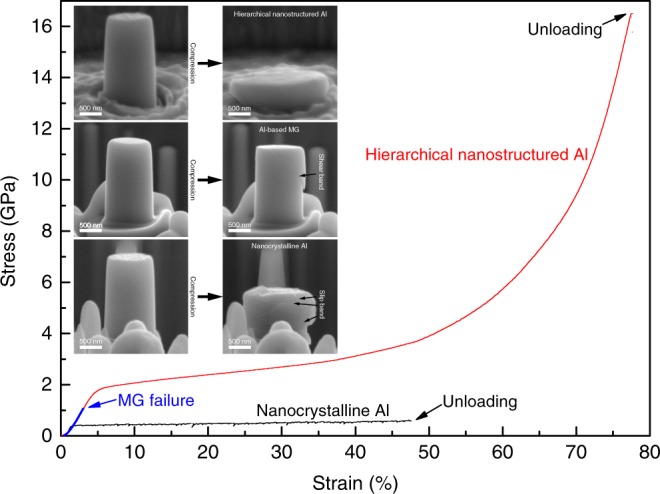
Mechanical property of the hierarchical nanostructured Al alloy. Compressive engineering stress-strain curves of the hierarchical nanostructured Al alloy, Al-based MG and nanocrystalline Al pillar samples with the same diameter of 1 μm. The insets are the SEM images of the samples before and after compression

The overall high strength and flexibility properties of the hierarchical nanostructured Al alloy are illustrated in Supplementary Movie 2. The alloy’s mechanical properties were analyzed by scanning electron microscope (SEM) in situ compression and tension tests under identical conditions (sample size and loading rate) for the hierarchical nanostructured Al alloy, Al-based MG and nanocrystalline Al. Compressive engineering stress-strain curves (Fig. 3) show that nanocrystalline Al has a yield strength of only 0.2 GPa with discontinuous plastic flow while Al-based MG has a yield strength of 1.0 GPa without any plasticity. The mechanical performance of both nanocrystalline Al and Al-based MG agrees well with previous reports^23,32^. In contrast, the hierarchical nanostructured Al alloy displays an ultrahigh yield strength of 1.7 GPa, and a homogeneous large plastic deformation with a strain of over 70%. Larger samples of the hierarchical nanostructured Al alloy (8 μm diameter) were tested to eliminate the possibility of a size effect (Supplementary Fig. 4). The sample size effect on mechanical properties usually appears when the size of the sample is comparable to its defect. There is a low probability of defects existing inside the small sample^33^, which induces a near-ideal strength^20,34^. It is believed that the 3 µm-diameter sample is able to exclude the size effect for a nanotwinned Al alloys^30^. Because the size of our 8 µm-diameter sample is more than 80 times larger than that of its structure unit, it may possess little sample size effect. After compression, nanocrystalline Al and Al-based MG reveal slip bands and shear bands, respectively, which are typical signatures of inhomogeneous deformation. However, the hierarchical nanostructured Al shows homogeneous deformation, which is confirmed by both SEM images of the pillar sample after compression and the SEM in situ compression movie (Supplementary Movie 3). Furthermore, in the SEM in situ tension test (Supplementary Fig. 5 and Movie 4), strength and ductility in tension are revealed. True stress and true strain are calculated by measuring the instantaneous load and gauge width^18^. The hierarchical nanostructured Al alloy shows a tensile yield strength of 1.2 GPa with 17% fracture strain (60% ultimate true strain). While nanocrystalline materials have relative higher strength than their coarse-grained counterparts, shear localization always takes place^32^. MGs have a much higher strength than their crystalline counterparts, yet inevitably display limited plasticity as a result of shear band instability^6^. By combining these two nanostructural units to form the hierarchical nanostructure, we can achieve ultrahigh strength with homogeneous deformation. The GBs usually act as dislocation sources for the nanocrystalline alloys^35,36^. In the hierarchical nanostructured Al alloy, however, the GBs are mostly replaced by the nano-sized MG phase. The extremely small-sized MG is believed to reach near-ideal strength^20^, which effectively impede the dislocation generation in the crystalline phase, thus enhancing the yield strength. The detailed plastic deformation mechanisms will be discussed further below. It is worthwhile to note that the focused ion beam (FIB) current was controlled to be as small as possible (1.5 pA at the final milling stage) to minimize the surface damage of the micro-pillar samples by ion beam. It is realized that the surface damage layer generated at 30 kV Ga FIB milling is 6 nm thick^37^ for Al alloys, which is thin compared to the diameter of the micro-pillar. Although it is reported that the surface damage effect may lead to yield strength decrease (less than 20% for 1 µm-sized pillar) of nanocrystalline Al, it does not influence plasticity^38^. In order to exclude the surface damage effect on the mechanical property of the glass-crystal hierarchical nanostructure in the specific alloy system, the same compression test was conducted on a hierarchical nanostructured Mg alloy micro-pillar (Supplementary Fig. 6). It also shows much higher strength and plasticity than that of its MG counterpart. Therefore, the hierarchical nanostructure overcomes strength-plasticity trade-off, which is a long-standing goal in structural materials research^39,40^. Furthermore, Al is an important light-weight structural material with extensive applications in different industries. By comparing specific yield strength vs. *E*/*σ*_*y*_ of the hierarchical nanostructured Al alloy with other ultrastrong materials (Supplementary Fig. 7), our study shows the achievement of ultrahigh specific strength by the hierarchical nanostructure.

## Discussion

From the compressive true stress-strain curve (Supplementary Fig. 8a), strain hardening exists from 70 to 150% true strain. Such strain hardening does not come from the lateral constraint effect due to the friction between indenter and the top surface of the pillar, which is usually seen in later stage compression^41^. To verify this, the strength of a pre-compressed larger pillar was tested. A larger pillar with a diameter of 2 μm and a height of 4 μm was compressed to about half of its original height, then was milled using FIB to have a 2:1 aspect-ratio, that is 1 μm-diameter pillar (illustrated in Supplementary Fig. 8b inset images). The compression experiment was conducted on this pillar again. The result shows a higher strength when compared with the same dimension pillar milled from the as-received sample, thus confirming the existence of strain hardening (Supplementary Fig. 8b).

**Fig. 4:**
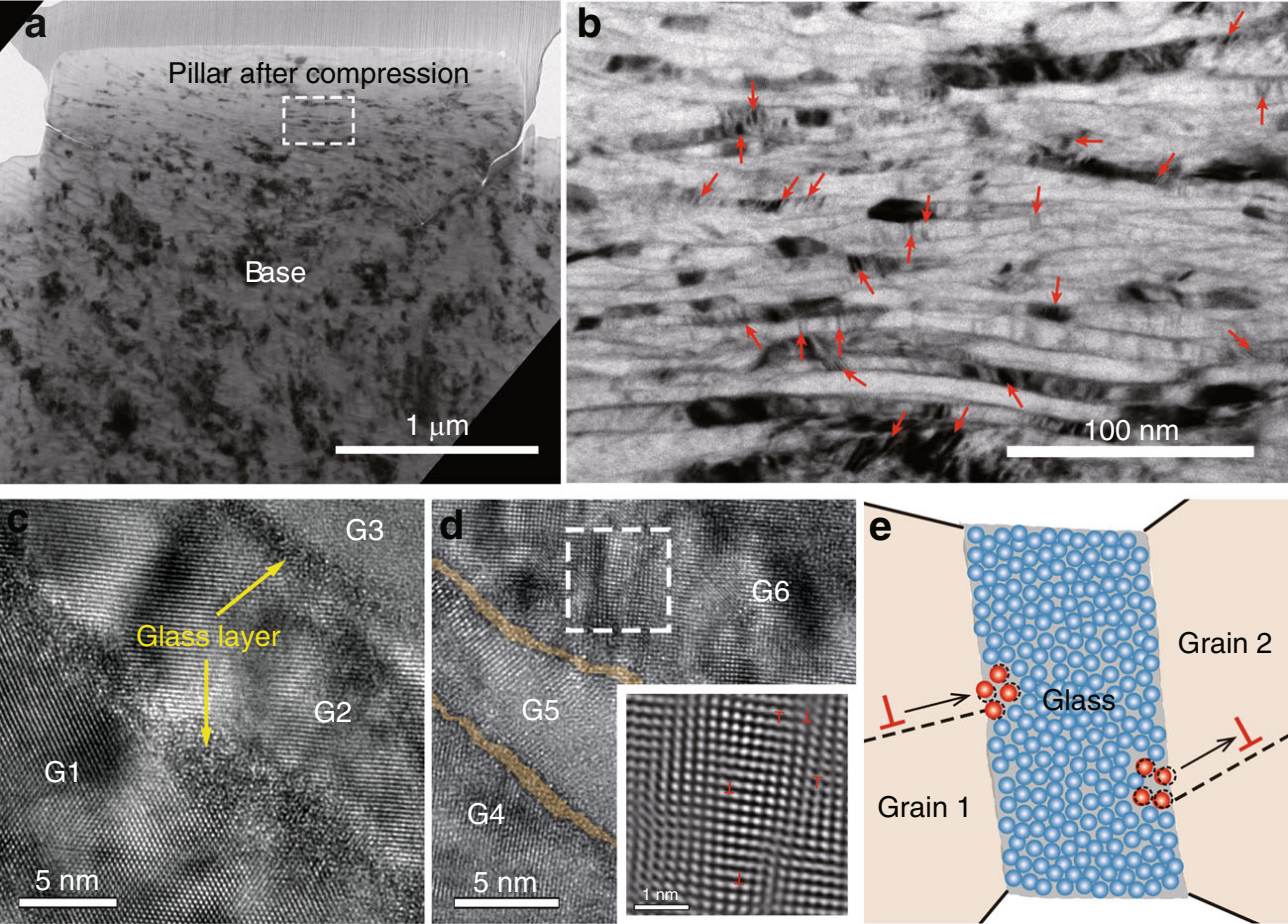
Plastic deformation mechanism of the hierarchical nanostructured Al alloy. **a** Cross-sectional TEM image of a 1 μm-diameter pillar after compression. **b** Enlarged TEM image from white dashed rectangle area in (**a**). The red arrows indicate the positions of some dark regions. **c** HRTEM image near nano-lamellar grains G1, G2, and G3, clearly demonstrates the existence of the amorphous phase (glass layer) after deformation. **d** HRTEM image near nano-lamellar grains G4, G5, and G6. The nano-sized MG phase is colored by light yellow. The lower right inset is inverse Fourier transformation (IFT) image of the dashed square area in the main image, showing some of the dislocations ‘_┴_’. **e** Illustration of dislocations’ activities interacted with the nano-sized MG phase. A dislocation (‘_┴_’) is generated on the glass-grain 2 interface and then moves inside grain 2. Another dislocation (‘_┴_’) moves inside grain 1 and then is absorbed by the atoms on the edge of the nano-sized MG phase (dislocation annihilation). The red and blue spheres represent mobile and less mobile atoms respectively. The dashed circles represent the original positions of the mobile atoms. The black arrows denote the motion directions of the dislocations

In tension tests, strain hardening appears from 10 to 55% true strain (Supplementary Fig. 5 and Movie 4), and appears in both the compression and the tension for the hierarchical nanostructured Al alloy. It is generally known that strain hardening has difficulty taking place in amorphous and nanocrystalline materials. The reason is that in amorphous materials, work softening due to the shear-dilation prevails during plastic deformation^6^, and in nanocrystalline materials, plastic deformation is usually associated with grain boundary (GB) activity^42,43^, such as grain growth, GB sliding or grain rotation that lead to strain softening. Therefore, the deformation mechanism of the hierarchical nanostructured Al alloy should be different from that of monolithic amorphous or nanocrystalline phases. To understand the deformation mechanism, the microstructure of the compressed pillar with a true strain of 150% (engineering strain of 76%) was investigated by TEM (Fig. 4). The overall compressed pillar shows a flat-barrel shape without any shear plane/offset (Fig. 4a), which indicates homogeneous plastic deformation. In the larger magnification TEM image of the compressed pillar (Fig. 4b), the nanograins show the lamellar structure, and the vertical width of the nanograins decreases from ~40 to ~ 8 nm after compression. Nano-lamellar grains orientated with crystallographically low-index planes (Fig. 4c) is helpful to reveal the extremely thin MG layer between them. It shows that the width of the nano-sized MG phase decrease from ~4 to ~1 nm after plastic deformation. This confirms the existence of fully homogeneous plastic flow of the nano-sized MG phase without any shear band^18^.

Previous investigations pointed out that due to thermally activated GB migration, grain refinement of low melting temperature metals by straining is extremely difficult^44^. The smallest grain size of 65 nm for Al was achieved by surface mechanical grinding treatment^44^. In our study, ~8 nm grain size was achieved in a compressed hierarchical nanostructured Al. In other words, the nano-sized MG phase in the hierarchical nanostructured Al effectively impedes GB migration, which prevents softening mechanisms (such as grain growth) from taking place. It is known that the softening of the conventional nanocrystalline materials originates from the instability of the GBs^45^. However, for the hierarchical nanostructured Al alloy, most of the GBs are replaced by a nano-sized MG phase, which impedes dislocations motion from one grain to another, contributing to the alloy’s ultrahigh strength. In the deformed sample, plenty of striped dark regions can be seen from the bright field (BF)-TEM image (Fig. 4b). These dark regions result from bending of the crystals to orientations closer to a good two-beam condition. In the HRTEM image (Fig. 4d), it is clear that the dark regions contain high-density dislocations, which are regarded as dislocations piled up inside nanograins. This mechanism contributes to the strain hardening of the nanograins. Furthermore, the homogeneous plastic flow in the nano-sized MG phase is notable, because bulk MGs usually exhibit limited plasticity due to shear-band instability. This phenomenon may be attributed to MGs’ intrinsic plastic deformation ability in extremely small size^18,19^. The flow stress of the nano-sized MG phase may reach to theoretical strength during plastic flow^18^. This indicates that the nano-sized MG phase is still strong during plastic deformation and is able to prevent the softening mechanism of the nanograins, such as GB migration in the conventional nanocrystalline materials. Moreover, its homogeneous flow behavior accommodates the large plastic deformation of the nanograins, which prevents shear deformation mechanism, such as stress localization. The interface usually plays a key role for crack deflection, thus enhancing toughness^46^. In this hierarchical nanostructured Al alloy, the MG phase on the interface has plastic deformation and no void or crack is seen in the highly deformed sample (Fig. 4), which may indicate a high toughness of the alloy.

To further demonstrate the dynamic structure evolution during compression, a TEM in situ compression experiment was performed on a nanopillar (Supplementary Movie 5). During plastic deformation, dislocations can be generated on the glass/crystal interfaces (dark contrasts denoted by yellow solid circles in Supplementary Movie 5) and move inside the grains. Some of them may pile up inside the grains, and some may move towards the nano-sized MG phase. Because the nano-sized MG phase can flow at such high stress level^18,19^, the mobile atoms on the edge of the MG phase are able to act as the ideal sink for dislocations when they are encountered. As a consequence, these dislocations disappear at the interfaces and the grain regions near the glass/crystal interfaces return to quasi-dislocation free (denoted by yellow dashed circles in Supplementary Movie 5). Since these kinds of dislocations temporarily appear during plastic deformation, they are termed transitory-dislocations. The continuous generation-movement-annihilation of transitory-dislocations (illustrated in Fig. 4e) contributes to the homogenous deformation of the hierarchical nanostructured Al alloy, and this mechanism is further revealed in an MD simulation (Supplementary Figs. 9–13). Similar dislocation movement in the nanograins also appears in a TEM in situ tension test (Supplementary Movie 6). The real-time Supplementary Movie 6 was captured after a tension pulse with a speed of 500 nm per second.

In summary, a hierarchical nanostructured Al alloy was developed with the assistance of computer simulation. The structure unit is a pair of ~40 nm-sized nanograins surrounded by ~4 nm-thick MG phase. The alloy achieves ultrahigh yield strength and large plasticity in both tension and compression. Its ultrahigh strength is a result of the nano-sized MG phase impeding dislocation gliding from one nanograin to another nanograin in the neighboring structure unit, while its large plasticity is a result of the continuous transitory-dislocations’ generation-movement-annihilation in the nanograins and the intrinsic plastic flow of the nano-sized MG phase. Our results illustrate a hierarchical nanostructure approach in material engineering and may contribute to not only the development of tough lightweight alloys but also the applications of micro-electromechanical systems (MEMS) flexible wearable devices.

## Methods

### Fabrication of the materials

We use magnetron sputtering as the fabrication method. We use an Al_92_Ni_2_Y_6_ (at.%) alloy target with the purity of 99.9%. Then the hierarchical nanostructured Al_95_Ni_2_Y_3_ (at.%) with the thickness of 17–50 µm were deposited on the Si (0 0 1) substrate. In the sputtering process, the Ar pressure was 0.2 Pa; the deposition rate was 12.5 nm per minute; the substrate bias voltage was −50 V. The Al_87_Ni_5_Y_8_ (at.%) MG was fabricated by melt-spun method. A pure Al plate with thickness of 1.5 mm was treated by surface mechanical attrition treatment to generate nanocrystalline grains on the surface. The nanocrystalline layer with uniform equiaxed grain size of ~300 nm has a thickness of ~50 μm. The 4 µm-thick Mg-based MG (Mg_58_Zn_37_Ca_5_ (at.%)) film was fabricated by magnetron sputtering using a Mg_60_Zn_35_Ca_5_ (at.%) alloy target with 99.9% purity. During the sputtering process, the Ar pressure was 0.2 Pa; the deposition rate was 10.6 nm per minute; the substrate bias voltage was −50 V. The Mg-based MG was then annealed at 300 °C to generate a glass-crystal hierarchical nanostructure. The thickness of the materials is large enough to fabricate micro-pillar samples by using focused ion beam (FIB).

### Structural characterization

The structure of the hierarchical nanostructured Al alloy was studied by TEM. We used a JEM 2100F FEG transmission electron microscope (from JEOL), operated at 200 kV, for TEM analysis. The thin-foil TEM samples were prepared with a FEI Scios™ DualBeam™ FIB, the final milling voltage/current was 2 kV/34 pA, which was small enough to avoid potential crystallization or amorphization. Needle-shaped specimens required for APT were fabricated by lift-outs and annular milled by FIB. The APT characterizations were performed in a local electrode atom probe (CAMEACA LEAP 5000XR). The specimens were analyzed at 50 K in voltage mode, a pulse repetition rate of 200 kHz, a pulse fraction of 20%, and an evaporation detection rate of 0.4% atom per pulse. Imago Visualization and Analysis Software (IVAS) version 3.8 was used for creating the 3D reconstructions and data analysis.

### Mechanical characterization

Nanoindentation was performed by Hysitron TI950 nanoindenter with a Berkovich tip. The indentation depth was kept below 10% of the film thickness to avoid the substrate effect. Micro-pillar and micro-dog-bone shaped samples were fabricated by FIB, with 30 kV/1.5 pA as the final milling condition. The height of the pillars was maintained smaller than the layer thickness. The aspect ratio (height/diameter) of the pillar was 2, and the taper angle of each nanopillar was less than 1.5°. Tension samples have square column cross-sections with gauge width of 2 μm, aspect ratio of 2. We conducted SEM in situ compression/tension tests at room temperature using a PI 85 PicoIndenter (Hysitron Inc.) with diamond punch/gripper inside a FEI Quanta 450 FEG scanning electron microscope, under displacement-control mode and at a strain rate of 1 × 10^−3^–5 × 10^−3^ s^−1^. During deformation before yielding in SEM in situ tension, the true stress and true strain were converted by using the equations:^18^ *σ*_T_ = *σ*_E_(1 + *ε*_E_) and *ε*_T_ = ln(1 + *ε*_E_), where *σ*_T_, *σ*_E_, *ε*_E_, and *ε*_T_ are the true stress, engineering stress, engineering strain, and true strain, respectively. After yielding, the instantaneous gauge width was measured from the in situ movie, and the true stress and true strain were calculated by using the equations:^1^ *σ*_T_ = *F*/*A*_i_ and *ε*_T_ = ln(*A*_0_/*A*_i_), where *A*_0_ and *A*_i_ are the gauge cross-sectional areas initial and during plastic deformation, respectively. TEM in situ uniaxial tension tests was conducted at room temperature using a Gatan 654 straining holder inside a JEM 2100F FEG transmission electron microscope (from JEOL), operated at 200 kV. TEM in situ compression tests was conducted at room temperature using PI 95 PicoIndenter (Hysitron Inc.) with 1 μm-diameter diamond punch inside the TEM, operated at 200 kV. The displacement rate was 2 nm per second.

### MD simulations

MD simulations were performed using LAMMPS package^47^ to study the binary Al-Ni system. The EAM potential developed by Pun et al.^48^ was employed to describe the atomic interactions within the Al-Ni system. With the consideration of the time scale limitation of MD simulation for nanocrystallization compared with the realistic formation process of nanometer-sized glass-crystal hierarchical nanostructure, we tried to construct the hierarchical nanostructured Al-based samples with the following process: a small cubic Al_85_Ni_15_ sample of (13,500 atoms) with periodic boundary conditions (PBCs) along all three dimensions is firstly equilibrated at 1800 K for 5 ns and then quenched to 300 K at a cooling rate of 10^11^ K/s, at 0 bar external pressure and under a Nosé-Hoover thermostat. Finally, the amorphous sample is relaxed at 300 K for 2 ns. We analyzed the equilibrated configuration of Al_85_Ni_15_. Supplementary Fig. 1a shows the variation of volume of Al_85_Ni_15_ from 1800 K to 300 K. The liquid to solid/glassy state transition point can clearly be observed by detecting the variation of volume during quenching. As observed from Supplementary Fig. 1b, the partial RDF curves of Al_85_Ni_15_ exhibits the main peaks of Al-Al, Al-Ni, and Ni-Ni at ~2.76 Å, ~2.55 Å and ~2.61 Å. These structure information is consistent with previous ab initio MD results^49,50^. Thus, the final relaxed configuration of Al_85_Ni_15_ could be a typical Al-based MG. For the mechanical test, a large sample is constructed by replications of the 13,500 atom configurations (~39.0 (x) × 39.0 (y) × 14.3 (z) nm^3^). Then the single crystalline pure-Al columnar grains with grain size about 10 nm is modeled and embedded into the quenched Al_85_Ni_15_ sample with a Voronoi diagram, controlling the thickness of Al_85_Ni_15_ about 3 nm. The sample contains about 1,320,000 atoms. This combined sample is further annealed for 0.5 ns before the tensile test. This annealing treatment is supposed to eliminate potential artifacts in the hierarchical nanostructured sample (Supplementary Fig. 1c). To compare the deformation mechanism between the hierarchical nanostructured Al-based alloy and the polycrystalline Al, a polycrystalline sample of Al is also built, with the same grain size, shapes, distributions, and lattice orientations as the hierarchical nanostructured one (Supplementary Fig. 1d). For compression loading, PBCs are imposed along three directions. A constant strain rate of ~2.5 × 10^8^ s^−1^ along *x-* or *y-*direction is imposed at a temperature of 300 K.

To monitor plastic shearing during mechanical deformation, the local Von Mises atomic shear strain^51^ in the samples is calculated. To carry out geometrical analysis on the atomic configurations, we have adopted various methods such as Voronoi tessellation method for local motif analysis, radial distribution function (RDF) for identification of amorphous phase, atomic coordination numbers (CNs) and common neighbor analysis (CNA)^52^, especially for crystal structure characterization.

Another model with crystalline phase size of ~25 nm and MG thickness of ~2.5 nm was designed, and uniaxial compression was simulated to evaluate the possible size effect on the plastic deformation of this Al-Ni dual-phase structure (Supplementary Fig. 2). In this simulated sample, the thickness of the MG phase approaches ~1/10^th^ of the crystalline phase, which is comparable to that of our experimental samples. Although the strain rates (~1.0 × 10^8^ s^−1^) in MD simulation are higher than that in experiments, it is generally accepted that simulation with the strain rate of this order is able to clarify the deformation mechanisms of the materials^53,54^. The atom configurations of the Al-Ni dual-phase sample before and after deformation are shown in Supplementary Fig. 2. The common neighbor analysis (CNA) and atomic shear strain coloring methods are employed. The dislocation-slip-based plastic deformation mode is clearly observed, similar to the results for a simulated sample with much larger fraction of MG phase (Fig. 1a–c). Furthermore, the vertical width of the grains decreases, which is consistent with our experimental results (Fig. 4).

The deformation modes of the hierarchical nanostructured Al-based alloy and nanocrystalline Al were further compared. ‘G1’ in Fig. 1b is selected and compared with the same grain in polycrystalline Al, at a strain of 7.5% (Supplementary Fig. 9). The length scale of grain along the compression direction changes from 10 to 8 nm (Supplementary Fig. 9b) in the hierarchical nanostructured Al-based alloy while it remains 10 nm in polycrystalline Al (Supplementary Fig. 9e) at a strain of 7.5%. In addition, similar dislocation propagation trajectories are observed in both of them (Supplementary Fig. 9c and f). It indicates that the interaction between dislocations and MGs are different from the traditional dislocation-grain boundary (GB) interaction, which account for the great ductility of our hierarchical nanostructured Al-based alloy. It should be emphasized that the length scale of grains gradually decreases along the compression direction, which could be an important factor for strengthening during plastic deformation since it is equivalent to grain refinement^55^.

In fact, the initiation of first dislocation, which represents the start of plastic deformation, occurs at a strain of ~5.5% and ~3.5% for the hierarchical nanostructured Al-based alloy and polycrystalline Al, respectively. That means the hierarchical nanostructured Al-based alloy can endure larger elastic stage, i.e., the initiation of dislocations from the glass/crystal interface is more difficult than polycrystalline Al, which could be evidence for the better mechanical stability of glass/crystal interface than GBs. In order to clearly identify the relationship between GBs and glass/crystal interface, a layered model was built (Supplementary Fig. 10a) and the deformation behavior under compression was simulated. Supplementary Fig. 10b–e show the successive snapshots of the initiation and propagation of dislocations from GBs. However, as a comparison, no dislocation initiates from glass/crystal interface, which indicates that the glass/crystal interface has better efficiency to impede dislocation motion than GB.

The interaction between dislocations and the MG phase was studied. The GB was replaced by Al_85_Ni_15_ MG in Supplementary Fig. 11a. It can be determined that the dislocations mainly nucleate from the glass/crystal interface, which has also been demonstrated in Supplementary Movie 1. Convex hulls (red circles in Supplementary Fig. 11b, c) appear on the glass/crystal interface, which results from the interaction between dislocations and the MG phase. The atomic shear strain caused by dislocations motion and MG flow can clearly be characterized in Supplementary Fig. 11e, f. More importantly, the regions pointed by orange arrows in Supplementary Fig. 11f are the MG regions that exhibit relatively large atomic shear strain. They are close to the positions where dislocation-interface interaction occurs. In contrast, larger atomic shear strain is not observed for the MG atoms close to the glass/crystal interface (the red dashed-rectangle region in Supplementary Fig. 11f), where no dislocation nucleation or accommodation occurs. In addition, Supplementary Fig. 12 directly shows that the roughness of the glass/crystal interface greatly increases after plastic deformation, which identifies the structural synergy during plastic deformation. Thus, these large atomic shear strain regions are mainly activated by the dislocation nucleation or accommodation, and the MG atoms can locally reconcile the atomic structure to endure large plastic deformation.

At last, the variation of crystal fraction was calculated after the compression in simulation (Supplementary Fig. 13). No matter compressing the sample along *x-* or *y-*direction, the fraction of crystal almost remains unchanged. This proves that no mechanical instability such as amorphization of Al grains and crystallization of Al_85_Ni_15_ MGs occurs, even at a large strain of 30%.

A hierarchical nanostructured Al-based alloy is studied by MD simulation and an extraordinary grain-flattening based plastic deformation behavior is successfully predicted. On one hand, dislocations nucleate more difficultly from the glass/crystal interface compared with the GB, which contribute to ultrahigh strength. On the other hand, a series of detailed MD studies help us to understand the origin of the exceptional properties of this hierarchical nanostructured Al-based alloy. The shear deformation is constrained at the glass/crystal interface and cannot cross the MG interior. Furthermore, the dislocation-based mechanism dominates the plastic deformation of the crystalline phase, which contributes to the large plasticity. The strong MG phase in the hierarchical nanostructured Al-based alloy impedes the large shear failure and well coordinate the plasticity by plastic flow.

## Supplementary information

Supplementary Information

Description of Additional Supplementary Files

Supplementary Movie 1

Supplementary Movie 2

Supplementary Movie 3

Supplementary Movie 4

Supplementary Movie 5

Supplementary Movie 6

## Data Availability

The data that support the findings of this study are available from the corresponding author upon reasonable request.
