# Supplementary material for: Hierarchical nanostructured aluminum alloy with ultrahigh strength and large plasticity
Source: Nat Commun. 2019 Nov 8;10:5099. doi: 10.1038/s41467-019-13087-4 (PMC6841713; doi:10.1038/s41467-019-13087-4)
Supplement: Supplementary file 1 — Supplementary Information [file 41467_2019_13087_MOESM1_ESM.pdf]

Hierarchical nanostructured aluminum alloy with ultrahigh strength and large plasticity -*Supplementary Information*

Wu, *et al.*

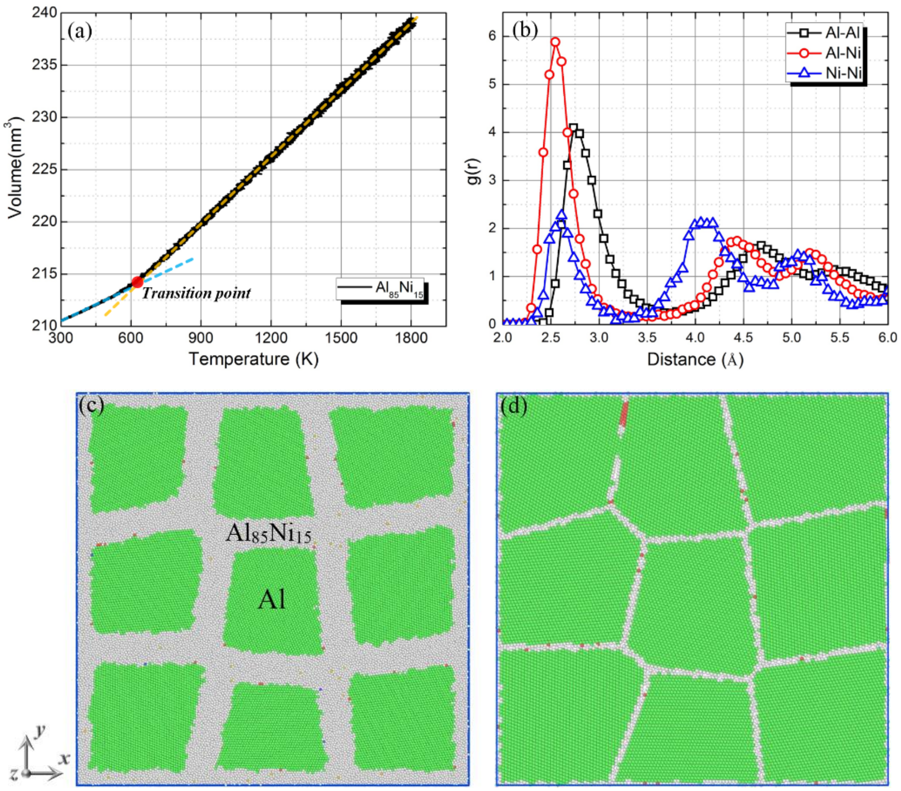

Supplementary Figure 1 | Structure of the hierarchical nanostructured Al alloy and polycrystalline Al in MD simulation. a, volume variation of $Al_{85}Ni_{15}$ during quenching. **b,** The partial RDF curves of $Al_{85}Ni_{15}$ after quenching and relaxation. The atomistic model of **c,** Glass-crystal hierarchical nanostructure composed of amorphous $Al_{85}Ni_{15}$ and Al nanograins, and **d,** polycrystalline Al, colored by CNA method (green: fcc, light grey: disordered).

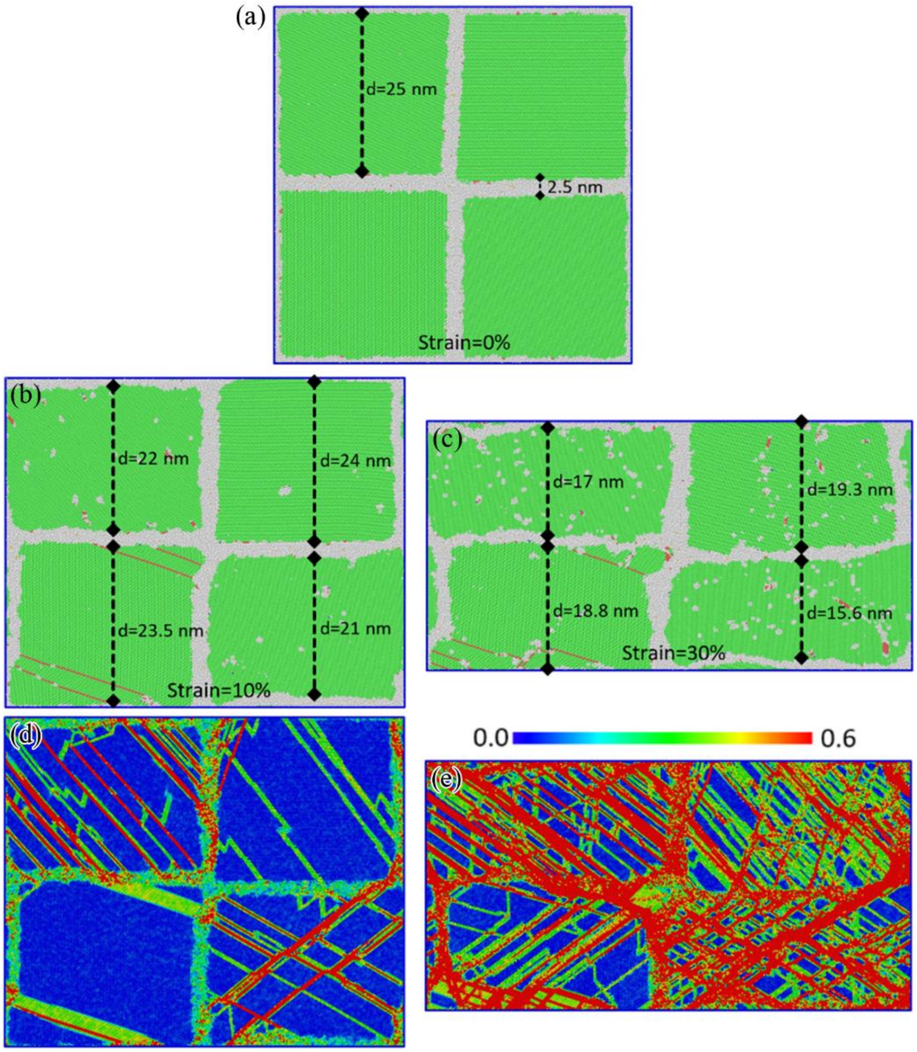

Supplementary Figure 2 | The atomistic model and deformation mode of the glass-crystal hierarchical nanostructure with 2.5 nm-thick MG phase. The atom configurations at strains of **a,** 0%, **b,** 10% and **c,** 30% colored by CNA method. The corresponding ASS distribution of the hierarchical nanostructure at the strain of **d,** 10% and **e,** 30%.

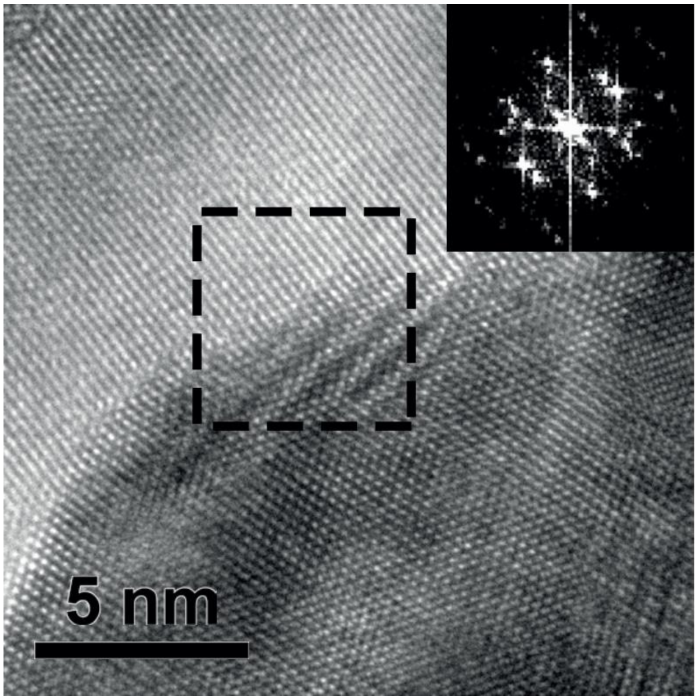

Supplementary Figure 3 | HRTEM image of the grain-grain interface in the crystallized Al alloy. The hierarchical nanostructured Al alloy was annealed at 300 ºC for 2 h to fully crystallize the amorphous phase. The inset FFT image is generated from the dashed rectangle area in the main image, showing this area is not amorphous.

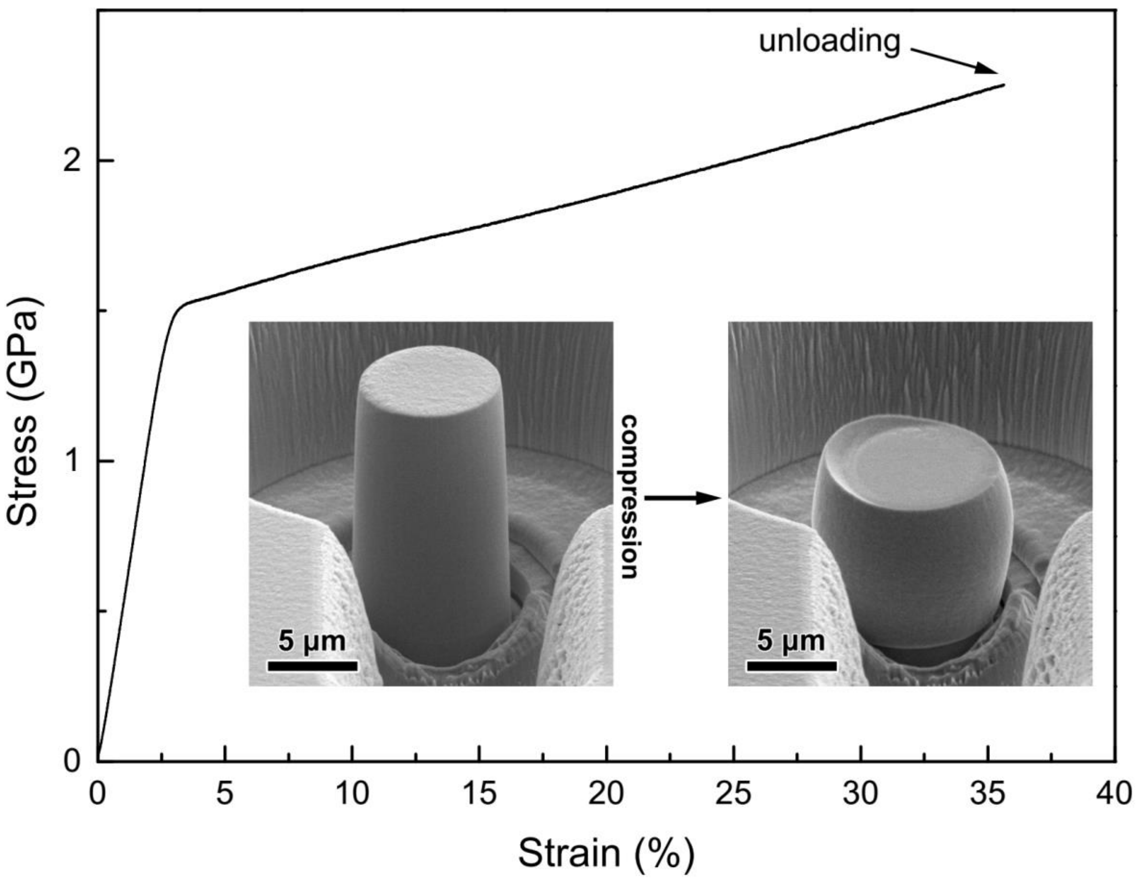

Supplementary Figure 4 | Mechnical property of the hierarchical nanostructured Al alloy pillar with diameter of 8 μm. The insets are SEM images (tilt by 60°) of the pillar before and after compression, which show homogeneous plastic deformation.

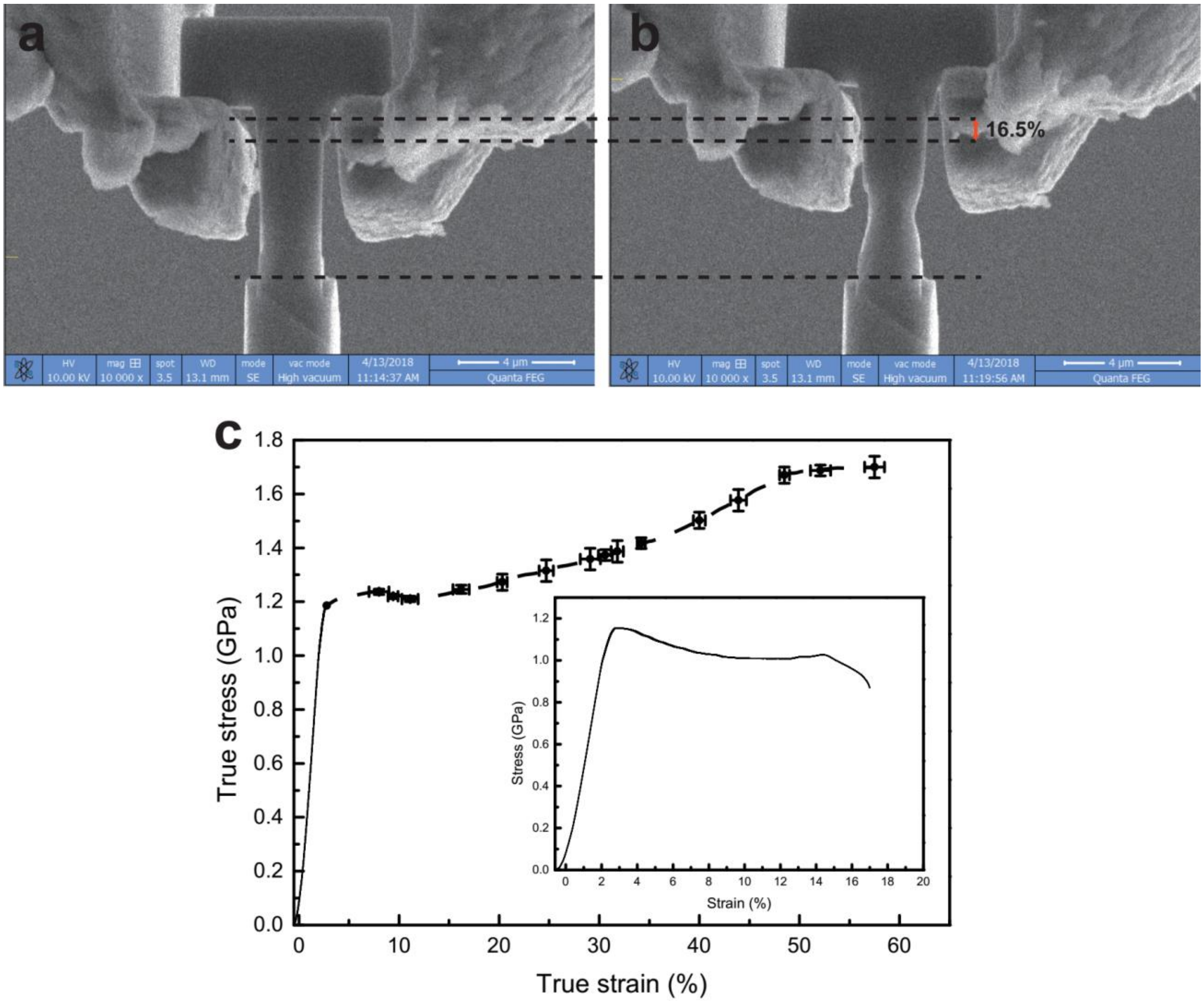

Supplementary Figure 5 | Tension property of the hierarchical nanostructured Al alloy (2 μm-width dog-bone shaped sample). Snapshots from SEM *in-situ* tension movie (Supplementary Movie 4) **a,** before and **b,** during tension, showing 16.5% engineering strain of the sample. **c,** Tensile true stress-strain and engineering stress-strain (inset) curves of the sample. The error bars are standard deviations.

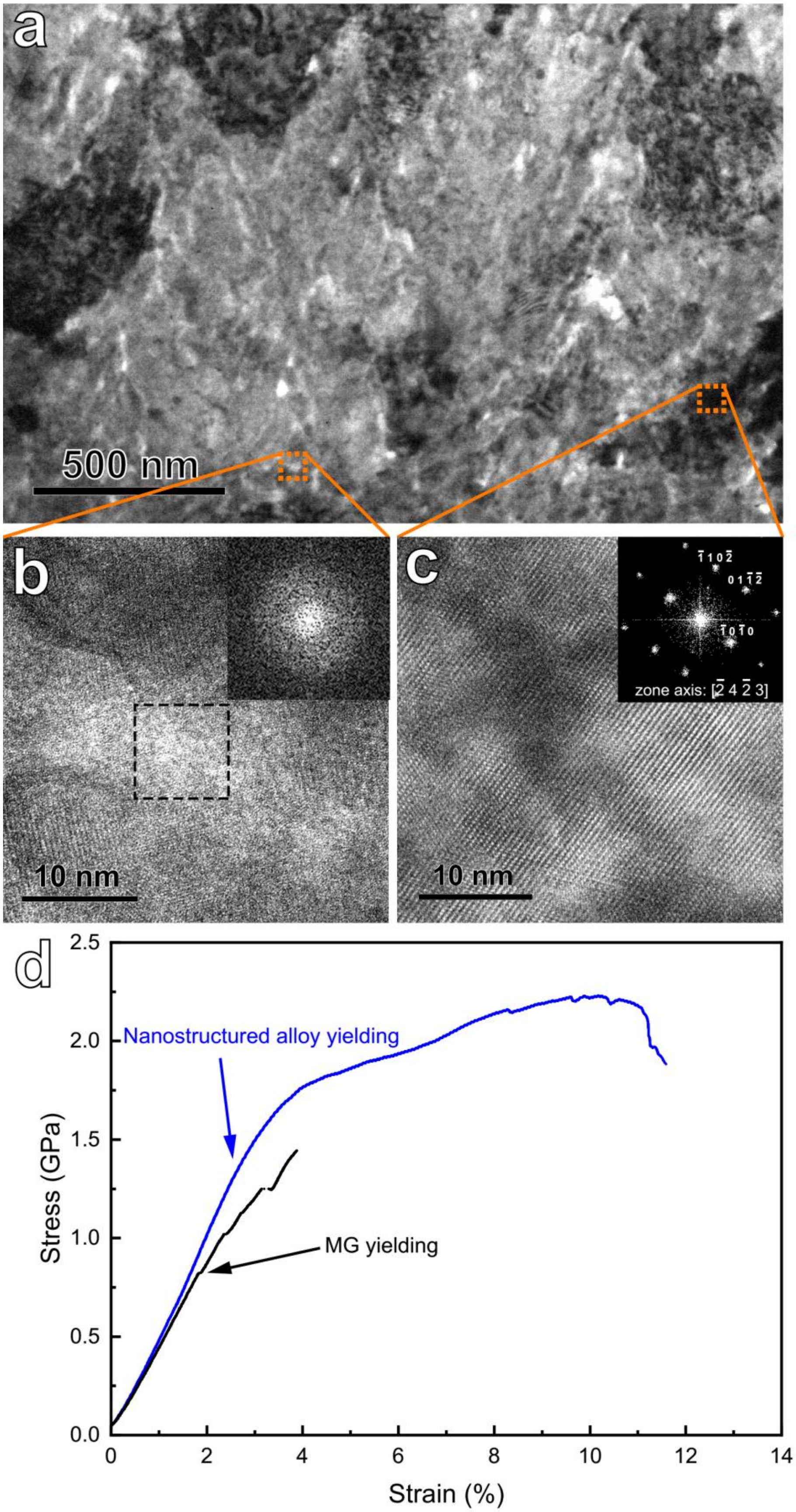
a
500 nm
b
10 nm
c
10 nm
zone axis: [2̄ 4 2̄ 3]
d
Nanostructured alloy yielding
MG yielding
Stress (GPa)
Strain (%)
0.0
0.5
1.0
1.5
2.0
2.5
0
2
4
6
8
10
12
14

Supplementary Figure 6 | Structure and mechanical property of the hierarchical nanostructured Mg alloy. a, Cross-sectional TEM image of the hierarchical nanostructured Mg alloy, showing nanograins surrounded with light contrast grain-grain interfaces. **b** and **c** are the zoomed-in images in **a**. **b** shows a nano-sized amorphous phase forms between the two grains. The FFT image (inset) of the dashed rectangle region in **b** shows a halo ring pattern, indicating an amorphous structure. **c** shows a *hcp* structure with zone axis of [-2 4 -2 3]. **d,** Compressive engineering stress-strain curves of the hierarchical nanostructured Mg alloy and Mg-based MG pillar samples with the same diameter of 1 μm. The plasticity of the hierarchical nanostructured Mg alloy is lower than that of the hierarchical nanostructured Al alloy, which may be attributed to that its nanograins possess *hcp* structure and are not homogeneously distributed.

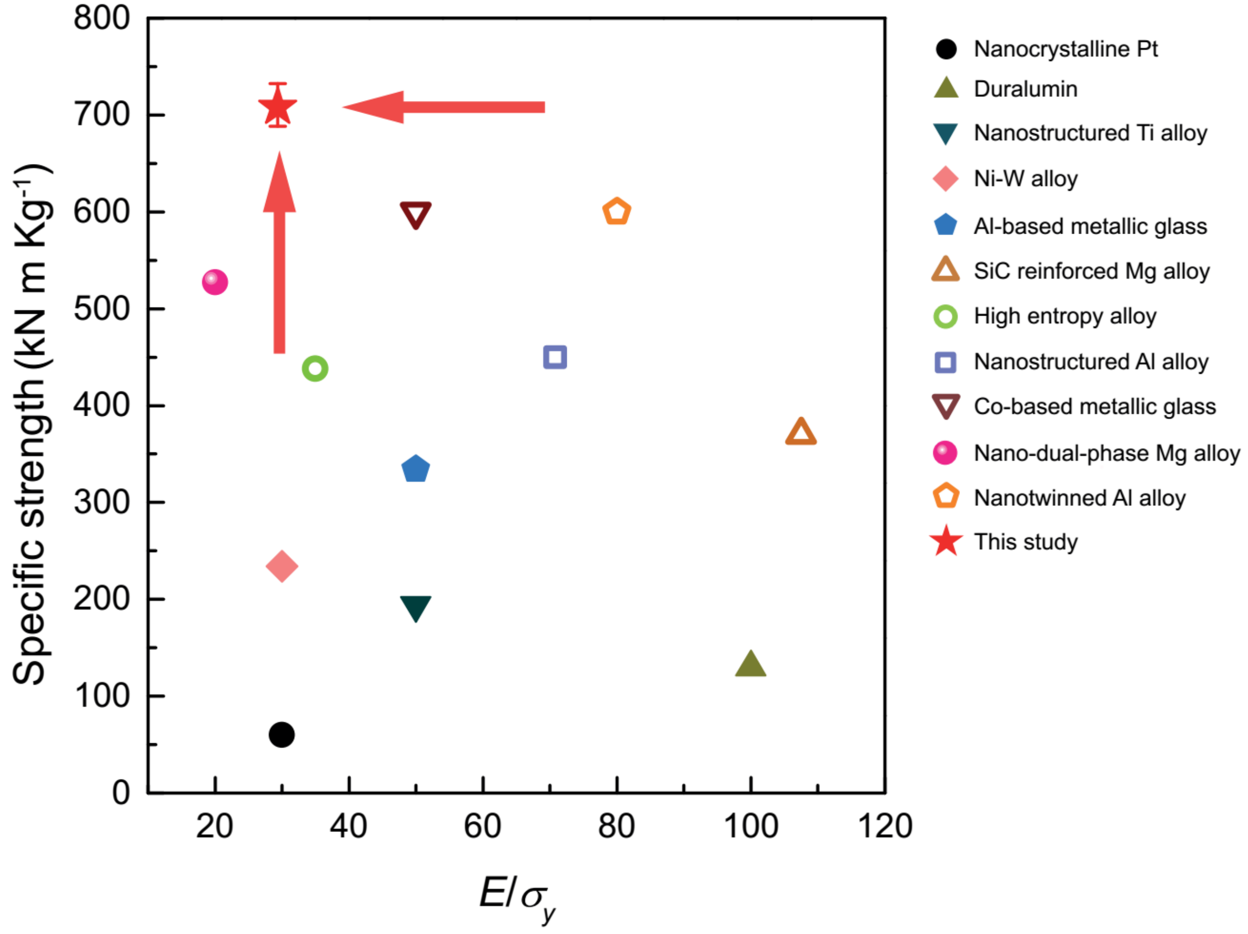

Supplementary Figure 7 | Specific yield strength vs. E/σ_y diagram for ultrastrong materials, where E is the Young's modulus and σ_y is the yield strength of a material. Smaller E/σ_y indicates the strength is much closer to the theoretical limit, that E/σ_y=20 is the near ideal-strength regime[14]. The data points are all from compression tests[1-11]. The error bar is standard deviation.

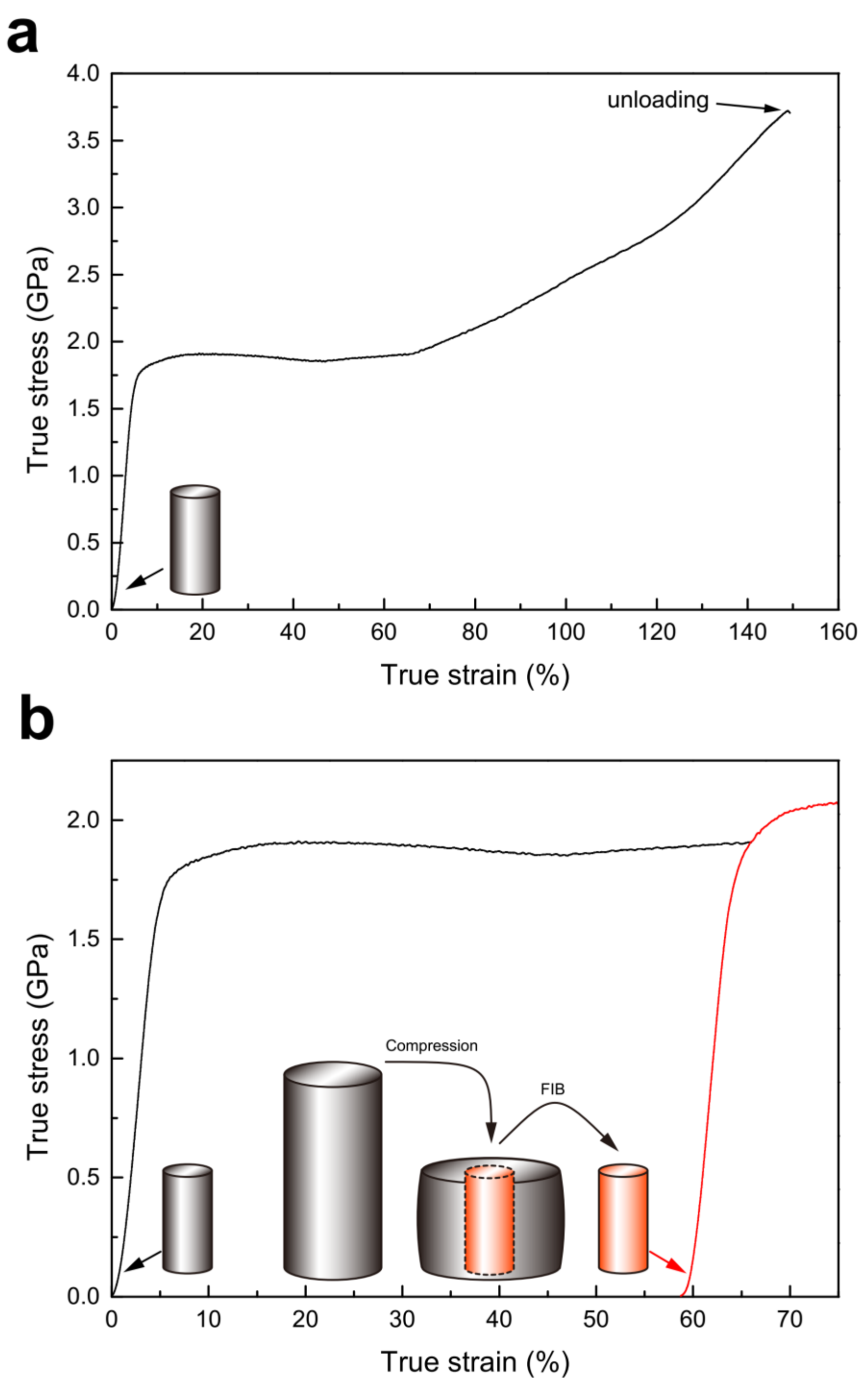

Supplementary Figure 8 | Compressive true stress-strain curve for the hierarchical nanostructured Al alloy pillar sample (1 μm diameter), showing **a,** ultrahigh strength and large plasticity with strain hardening. **b,** a 2 μm-diameter pillar sample was compressed to 66% (dark line), and subsequently was milled to diameter of 1 μm with aspect ratio of 2:1, and at last was performed by compression test (red line), which confirms the strain hardening capacity. The inset images in **a** and **b** illustrate relative size of the pillar samples and their fabrication procedure.

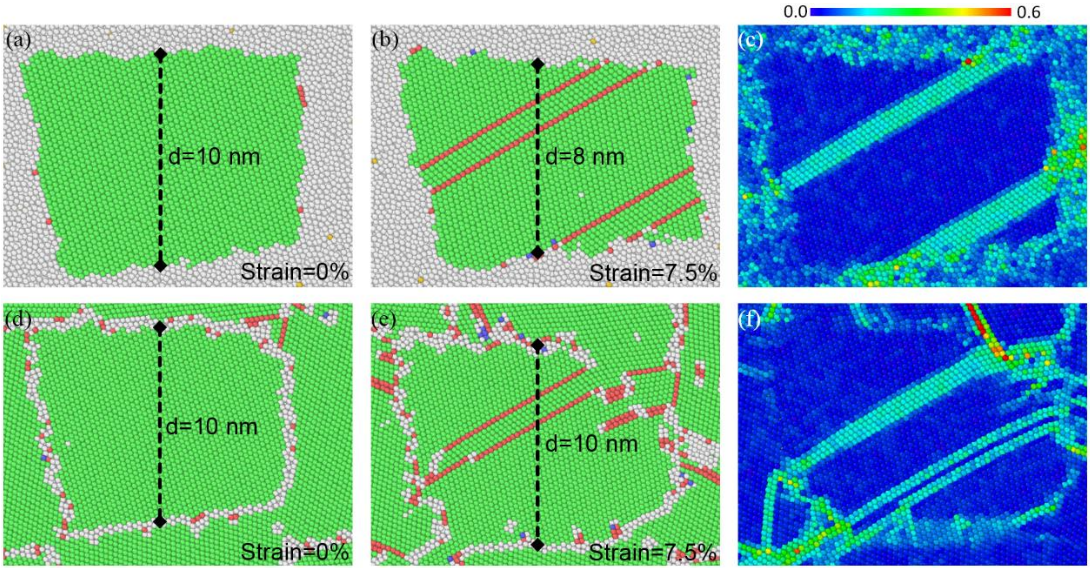

Supplementary Figure 9 | Structure evolution difference between the hierarchical nanostructured Al alloy and polycrystalline Al in MD simulation. The atomic configurations of the hierarchical nanostructured Al-based alloy (**a** and **b**) and polycrystalline Al (**d** and **e**) at a strain of 0% and 7.5%, colored by CNA method. The corresponding ASS configurations at a strain of 7.5% for the hierarchical nanostructured Al-based alloy (**c**) and polycrystalline Al (**f**).

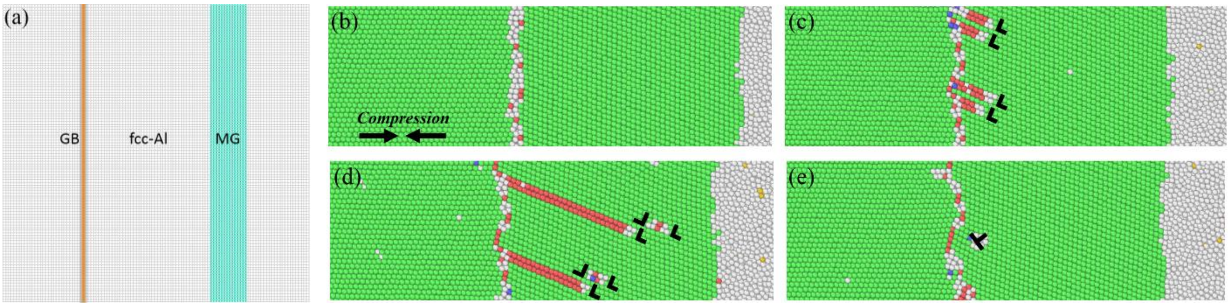

Supplementary Figure 10 | Structure evolution of a layered model fcc-Al/GB/$Al_{85}Ni_{15}$ MG in MD simulation. a, The schematic diagram. **b-e,** The successive atomic snapshots during compression.

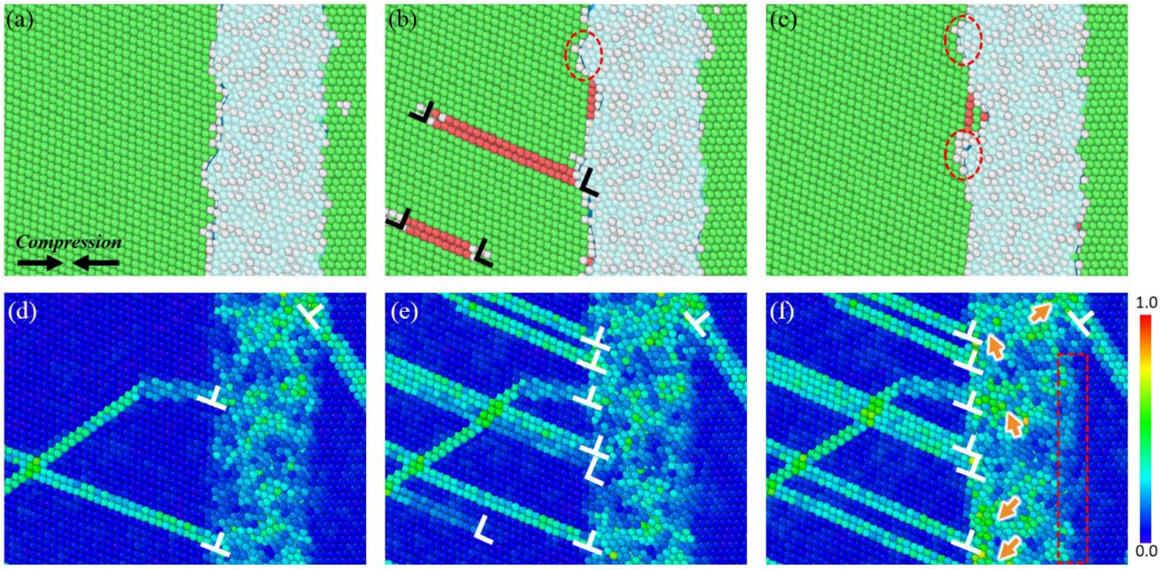

Supplementary Figure 11 | Structure evolution of a layered model fcc-Al/$Al_{85}Ni_{15}$ MG in MD simulation. The successive atomic snapshots colored by CAN method (**a-c**) and the corresponding ASS configurations (**d-f**) during compression.

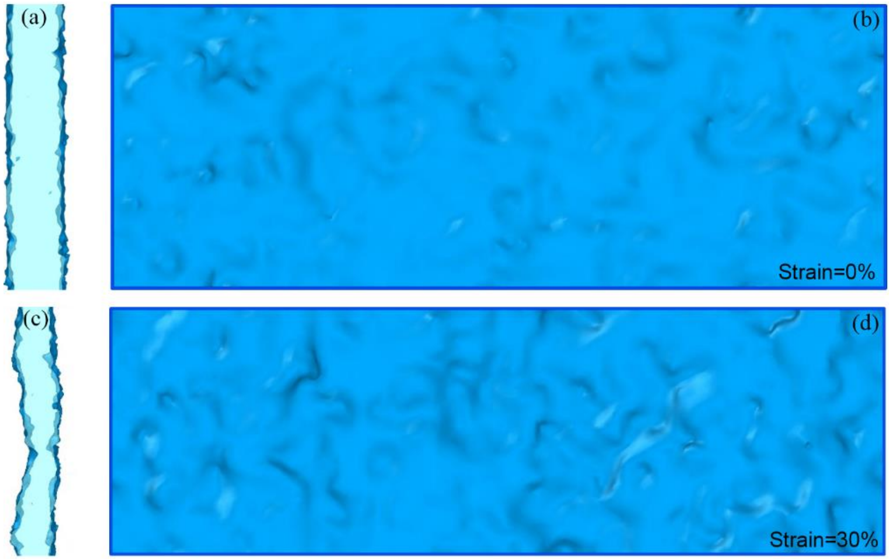

Supplementary Figure 12 | Morphology evolution of the glass/crystal interface in MD simulation. Front view and side view of the glass/crystal interface morphologies in a layered model fcc-Al/$Al_{85}Ni_{15}$ MG before (**a** and **b**) and after (**c** and **d**) compression.

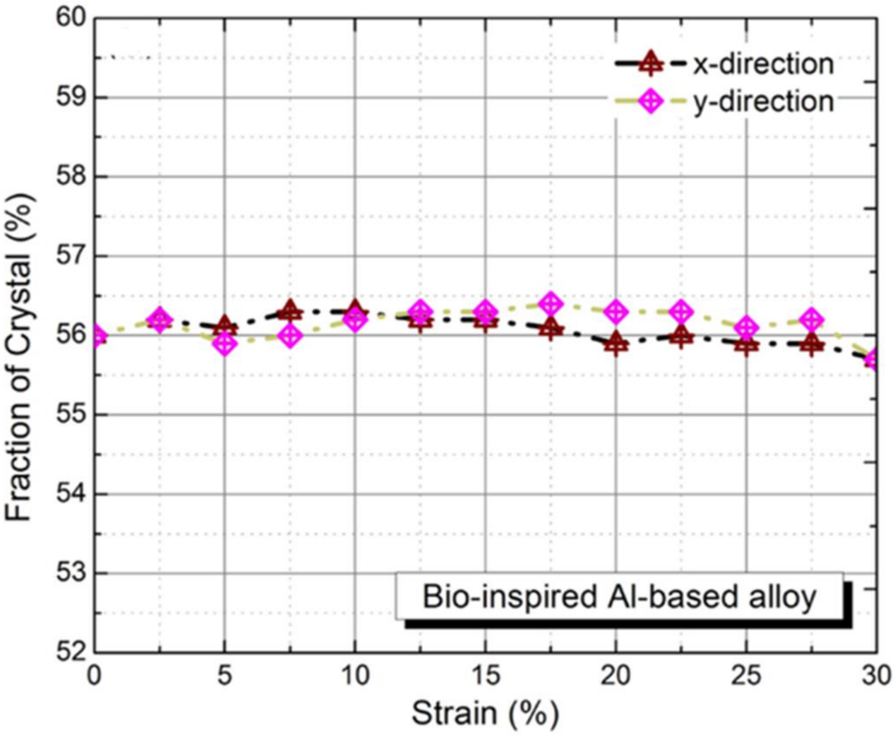

Supplementary Figure 13 | The variation of the fraction of crystal in the hierarchical nanostructured Al-based alloy during plastic deformation in MD simulation.

Supplementary References

1 Wu, G., Chan, K.-C., Zhu, L., Sun, L. & Lu, J. Dual-phase nanostructuring as a route to high-strength magnesium alloys. *Nature* **545**, 80 (2017).

2 Li, Q. *et al.* High-Strength Nanotwinned Al Alloys with 9R Phase. *Adv. Mater.* **30**, 1704629 (2018).

3 Inoue, A., Shen, B., Koshiba, H., Kato, H. & Yavari, A. R. Cobalt-based bulk glassy alloy with ultrahigh strength and soft magnetic properties. *Nat. Mater.* **2**, 661 (2003).

4 Yang, B. *et al.* Al-rich bulk metallic glasses with plasticity and ultrahigh specific strength. *Scripta Mater.* **61**, 423-426 (2009).

5 Gu, R. & Ngan, A. Size effect on the deformation behavior of duralumin micropillars. *Scripta Mater.* **68**, 861-864 (2013).

6 Khalajhedayati, A. & Rupert, T. J. Emergence of localized plasticity and failure through shear banding during microcompression of a nanocrystalline alloy. *Acta Mater.* **65**, 326-337 (2014).

7 Sun, B. *et al.* Ultrafine composite microstructure in a bulk Ti alloy for high strength, strain hardening and tensile ductility. *Acta Mater.* **54**, 1349-1357 (2006).

8 Wang, Z. *et al.* Hybrid nanostructured aluminum alloy with super-high strength. *NPG Asia Mater.* **7**, e229 (2015).

9 Zou, Y., Ma, H. & Spolenak, R. Ultrastrong ductile and stable high-entropy alloys at small scales. *Nat. Commun.* **6**, 7748 (2015).

10 Gu, X. W. *et al.* Size-dependent deformation of nanocrystalline Pt nanopillars. *Nano Lett.* **12**, 6385-6392 (2012).

11 Chen, L.-Y. *et al.* Processing and properties of magnesium containing a dense uniform dispersion of nanoparticles. *Nature* **528**, 539 (2015).
