# Supplementary material for: Hierarchical nanostructured aluminum alloy with ultrahigh strength and large plasticity
Source: Nat Commun. 2019 Nov 8;10:5099. doi: 10.1038/s41467-019-13087-4 (PMC6841713; doi:10.1038/s41467-019-13087-4)
Supplement: Supplementary file 2 — Description of Additional Supplementary Files [file 41467_2019_13087_MOESM2_ESM.docx]

**Description of Additional Supplementary Files**

**File Name: Supplementary Movie 1**

**Description:** MD movie

**File Name: Supplementary Movie 2**

**Description:** overall flexible and tough properties

**File Name: Supplementary Movie 3**

**Description:** SEM in-situ compression (8× speed)

**File Name: Supplementary Movie 4**

**Description:** SEM in-situ tension (8× speed)

**File Name: Supplementary Movie 5**

**Description:** TEM in-situ compression (0.25× speed)

**File Name: Supplementary Movie 6**

**Description:** TEM in-situ tension (real time)
